# Hearing Loss in Neuromyelitis Optica Spectrum Disorder: Case Report and Systematic Review

**DOI:** 10.3390/jcm15020422

**Published:** 2026-01-06

**Authors:** Stefania Kalampokini, Effrosyni Koutsouraki, George Psillas, Effrosyni Karatzioula, Korina Kaffe, Martha Spilioti, Vasilios Kimiskidis

**Affiliations:** 1 1st Department of Neurology, AHEPA University Hospital, Aristotle University, 54636 Thessaloniki, Greece; ekoutsou@auth.gr (E.K.); froso-k@hotmail.com (E.K.); korinakaffe@gmail.com (K.K.); marthags@auth.gr (M.S.); kimiskid@auth.gr (V.K.); 2 1st Academic ENT Department, AHEPA University Hospital, Aristotle University of Thessaloniki, 54636 Thessaloniki, Greece; gpsyllas@auth.gr

**Keywords:** neuromyelitis optica spectrum disorder, NMOSD, deafness, hearing loss, plasmapheresis

## Abstract

**Background:** Sudden, non-traumatic hearing loss has been associated with vascular or inflammatory disorders. Hearing loss in Neuromyelitis optica spectrum disorder (NMOSD) is a very rare presentation. **Methods:** In this paper, we describe the case of a 58-year-old female patient with aquaporin-4-positive NMOSD exhibiting bilateral tinnitus and right-sided deafness in the context of a relapse. The auditory brainstem responses pointed to a lesion of the right peripheral auditory pathway (cochlea and/or auditory nerve). The patient’s hearing failed to improve after high-dose intravenous steroids; however, it showed slight improvement after plasmapheresis. We also conducted a systematic literature review in databases MEDLINE and Scopus in English, searching for all reported cases of hearing loss in NMOSD. **Results:** We included 10 studies reporting 15 cases of NMOSD with hearing loss. The vast majority of patients were female (11 out of 15, 73.3%), with an age range of 26 to 70 years. Hearing loss, ranging from mild to severe, seems more frequent in AQP4-positive cases, and it can even be the presenting symptom. It can present isolated or in combination with tinnitus, ataxia, and/or intractable vomiting. The auditory pathway impairment in NMOSD seems to be localized either centrally, i.e., cochlear nuclei or higher brainstem levels, or peripherally, i.e., in the cochlea or cochlear nerve itself. Intravenous methylprednisolone in high doses, followed by oral tapering, was the most common treatment option, resulting in a gradual improvement. **Conclusions:** This paper describes a rare case of peripheral auditory pathway affection in NMOSD, which is an inflammatory astrocytopathy mainly affecting the central nervous system. Early recognition of hearing loss in the context of an NMOSD relapse and subsequent treatment have a crucial impact on the hearing outcome of NMOSD patients. This expands our knowledge of NMOSD as an autoimmune aquaporin-4 channelopathy.

## 1. Introduction

The term “neuromyelitis optica spectrum disorder” (NMOSD) refers to an inflammatory astrocytopathy associated, in the vast majority of cases, with antibodies targeting aquaporin-4 (AQ4) [1]. Aquaporin-4 is a transmembrane channel protein, located in the astrocytic foot processes at the blood–brain barrier (BBB), capable of transporting water and small solutes across cells [1,2]. Common clinical presentations in NMOSD comprise severe uni- or bilateral optic neuritis, myelitis, and/or brainstem encephalitis, often presenting with intractable nausea/vomiting [1,3]. NMOSD usually follows a relapsing course without marked progression of disability between relapses, but in a minority of cases, the disease course can be monophasic, especially in AQ4-negative cases [4]. Typical magnetic resonance NMOSD findings include longitudinally extensive optic neuritis affecting the optic chiasm, longitudinal extensive cervico-bulbar myelitis affecting area postrema in the dorsal brainstem, diencephalic or periaqueductal gray matter lesions, extensive hemispheric lesions including periependymal lesions surrounding the lateral ventricles, as well as deep white-matter lesions [1].

Aquaporins comprise a family of membrane proteins with 13 different isoforms (AQ1 to AQ12) that transport water and small molecules (e.g., urea, glycerol) through the cell membrane [5,6]. AQ4, along with AQP1, is the most abundant aquaporin in the central nervous system (CNS) and is found in diverse areas throughout the brain, including the cerebral cortex, corpus callosum, retina, cerebellum, hypothalamus, and brainstem [7]. In the CNS, aquaporins are involved in signal transduction, astrocyte migration, and the development and maintenance of the BBB [7]. Furthermore, aquaporins play a crucial role in auditory physiology, as well as in various pathological processes that affect cochlear cells [8]. Among the nine aquaporins expressed in the inner ear (AQP1 to AQP9), AQP4 is known to play an important role in fluid homeostasis (endolymph and perilymph) of the inner ear [9,10]. AQ4 is expressed in the epithelial cells of the organ of Corti in the cochlea of the inner ear and participates in auditory formation and perception by maintaining the osmotic balance of ions, particularly potassium, required for hearing and balancing sensory excitability [8,11].

Sudden (non-traumatic) hearing loss has been associated with vascular or inflammatory disorders, i.e., viral infections or autoimmune disorders such as rheumatoid arthritis, Sjögren’s syndrome, anti-phospholipid syndrome, Wegener’s granulomatosis, and multiple sclerosis, although a large number of cases remain without any identifiable etiology [12,13]. It has been recently demonstrated that hearing loss is not uncommon in NMOSD and may be a prodromal or clinical symptom of either central or peripheral lesions, especially affecting the high-frequency range [14,15,16,17,18,19,20,21,22]. NMOSD lesions in cases of hearing loss are typically localized at cochlear and vestibular nuclei in the brainstem, which represent sites of high AQP4 expression [23]. However, some NMOSD cases presented hearing loss due to an extraaxial lesion of the vestibulocochlear nerve [17].

In this paper, we describe a case of a female patient with AQ4-positive NMOSD exhibiting bilateral tinnitus and right-sided deafness in the context of a relapse. Additionally, we summarize all previously reported NMOSD cases with hearing loss from the literature, i.e., their demographic, clinical, neurophysiological, and radiological characteristics and their treatment and outcome, and we discuss the underlying pathophysiology.

## 2. Case Presentation

A 58-year-old female patient with a medical history of aquaporin-4-positive NMOSD for 25 years, hydrocephalus treated with a ventriculoperitoneal shunt 20 years ago, hypertension, and hyperlipidemia, presented to the emergency department due to dizziness (vertigo), nausea, gait instability, tinnitus, and loss of hearing in the right ear for a few days. She was diagnosed with complete sensorineural hearing loss by an otolaryngologist in the outpatient setting. She was treated with methylprednisolone 8 mg bd and valaciclovir 500 mg bd, although no signs of herpes vesicles were present, without any improvement. She was under treatment with rituximab 1000 mg every 6 months for the last three years. The patient was admitted to the neurological clinic for further evaluation and treatment. Routine laboratory workup, anti-nuclear antibodies, rheumatoid factor, anti-Ro, and anti-La antibodies were negative. MOG antibodies were negative in serum, whereas AQP4 antibodies were positive, measured by a cell-based assay. The patient did not receive any ototoxic drugs.

**Figure 1:**
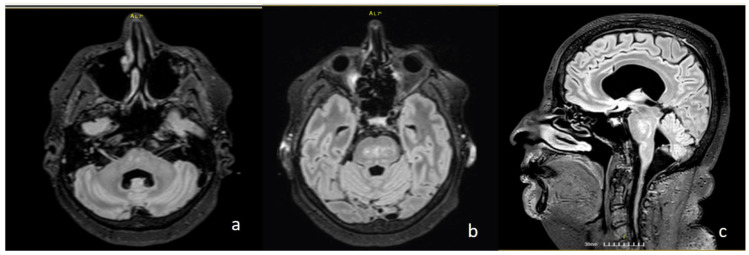
FLAIR images of the patient showing multiple non-enhancing lesions, mainly in the pons, (**a**) at the level of the vestibulocochlear nerve and nuclei, (**b**) at the level of the pons, and (**c**) sagittal view.

**Figure 2:**
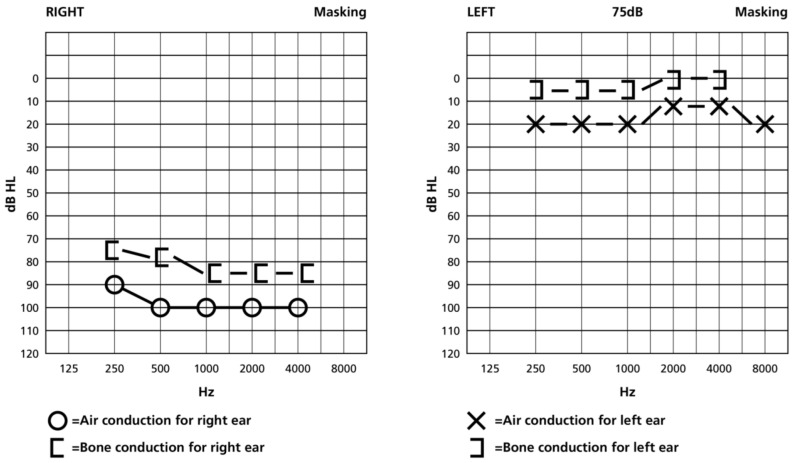
Pure-tone audiogram showing hearing threshold at 90–100 dBnHL at all frequencies 2.5–8 kHz, compatible with deafness/cophosis on the right.

The neurological examination revealed severe truncal ataxia, due to which she was not able to stand or walk. There was an absent pupillary light reflex with only light perception on the left eye and mild left spastic hemiparesis with hemihypesthesia, residual from previous NMOSD attacks. Magnetic resonance imaging (MRI) revealed multiple non-enhancing periventricular and subcortical lesions, as well as lesions in the pons (Figure 1). Hearing loss was quantified by a pure-tone audiogram, which showed hearing threshold at 90–100 dBnHL at all frequencies 2.5–8 kHz, compatible with deafness/cophosis (Figure 2). The auditory brainstem responses (ABRs) were normal on the left, while on the right, no waves I-V could be recorded (Supplementary Figure S1).

**Figure 3:**
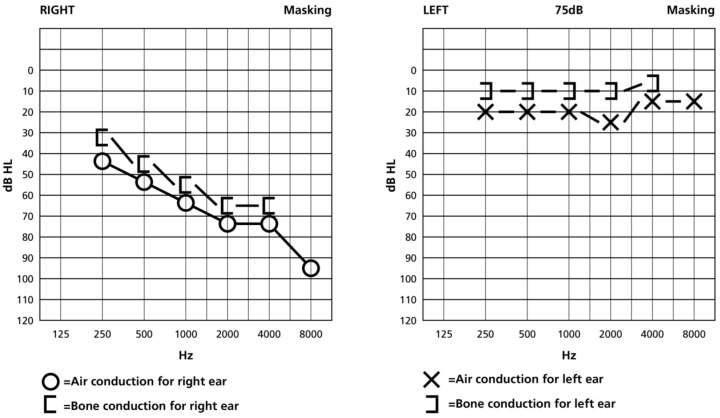
Pure-tone audiogram at 3-month follow-up, showing a downsloping pattern of hearing loss, from moderate hearing levels (45–55 dBnHL) at low frequencies to severe hearing levels (75–95 dBnHL) at high frequencies.

The patient received 1 g methylprednisolone/day for 5 days with mild improvement of ataxia but no improvement of hearing. The patient then received three intratympanic steroid injections (dexamethasone 4 mg each), but there was, again, no improvement in hearing. Subsequently, she underwent plasmapheresis q.o.d. for 14 days. A new pure-tone audiogram, after completion of plasmapheresis, showed slight improvement of hearing of the right ear. In particular, it showed severe sensorineural hearing loss at all frequencies, except for 8 kHz, where the hearing threshold was at 105 dBnHL (Supplementary Figure S2). After completion of meningococcal vaccination, the patient was started on ravulizumab 3000 mg as an initial dose and 3600 mg as maintenance every 8 weeks (body weight > 100 kg). At 3-month follow-up, her pure-tone audiogram showed a downsloping pattern of hearing loss, from moderate hearing levels (45–55 dBnHL) at low frequencies to severe hearing levels (75–95 dBnHL) at high frequencies (Figure 3). Clinical information of this case is summarized in Table 1.

## 3. Methods

We conducted a systematic literature review in databases MEDLINE and Scopus in all records till November 2025, following the PRISMA guidelines for Systematic Reviews [24]. The search strategy was as follows: (((deafness) OR (cophosis)) OR (hearing loss)) AND (((NMOSD) OR (neuromyelitis optica)) OR (neuromyelitis optica spectrum disorder)). Inclusion criteria were English language, original studies/case reports, or series. Exclusion criteria were articles not written in English, articles that involved laboratory animals, and non-original studies (reviews, editorials, expert opinions). No better explanation for the hearing loss should have been present, i.e., papers with co-existing conditions were excluded. Reference lists of the retrieved records were checked for additional potentially eligible studies. The full texts of eligible articles were reviewed by two independent authors (SK, Eko) to assess their relevance to the research question. Disagreements were discussed and resolved by consensus. From each eligible paper, the following information was extracted: age, gender, symptoms, type of hearing loss, MRI findings, audiogram or other neurophysiology findings, presence of AQ4 antibodies, treatment, and outcome. The quality of eligible studies was evaluated using the Joanna Briggs Institute (JBI) critical appraisal tools [25]. The JBI Checklist for case reports critically appraises the methodological quality of a study and assesses the potential for bias in its design, conduct, and analysis using eight items.

## 4. Results

**Figure 4:**
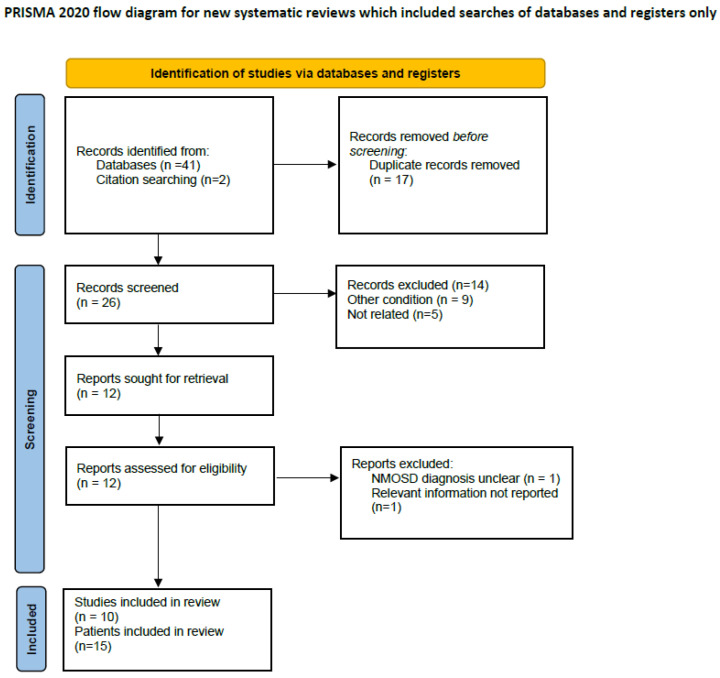
Flow chart of the included studies.

The initial search of databases yielded 41 studies, of which 17 were duplicates, and two additional studies were detected by hand search. From the 26 records, after exclusion of 14 studies referring to another condition or not related studies, based on screening of title and abstract, 12 studies were assessed for eligibility (full text was screened). From those, after excluding two studies (one in which the NMOSD diagnosis was not clear and one in which essential information was not reported), 10 studies including 15 cases of NMOSD with hearing loss remained for inclusion in the systematic review. All the included studies were of high quality (6 or higher). The flow chart of the included studies can be seen in Figure 4. As Supplementary Material you can find the PRISMA checklist. The demographic, clinical, radiological, and neurophysiological characteristics, the presence of AQ4 antibodies, the received treatment, as well as outcome measures concerning hearing can be seen in Table 2.

The included studies were 10 in total, dating from 2013 to 2024 [14,15,16,17,18,19,20,21,22,26]. The vast majority of patients were female (11 out of 15, 73.3%); there was only one male, while in 3 cases, gender was not reported. Age ranged from 26 to 70 years. NMOSD was associated with AQ4-positive antibodies in the vast majority of cases (12 out of 15, 80%). Hearing loss was accompanied by tinnitus in 5 patients [14,15,16,17,22], vertigo in three [15,17,22], ataxia in one [14], and nausea/vomiting in another one [15]. Isolated hearing loss was reported in six cases [19,20,21,22]. The type of hearing loss was sensorineural in most cases (11 out of 15, 73.3%), conductive or mixed in one case each, whereas in four cases, the type of hearing loss was not specified [20,21].

MRI of the brain showed lesions in the dorsal medulla, in proximity to the cochlear nuclei, in only three cases [14,19,26], while enlargement of the eighth cranial nerve with gadolinium enhancement was reported in a single case [17]. Audiogram was not performed or not reported in many instances; in those reported, high-frequency or low-mid frequency loss was recorded [15,16,17]. Auditory Brainstem Response (ABR) evoked potentials were performed in very few cases [14,16,18,19], with most of those being consistent with a central lesion.

With regard to treatment, methylprednisolone at high doses (1 g/day) for 5–12 days, followed by oral tapering for up to two months, was the most common treatment option, which yielded positive results in most cases [15,16,17,18,19,20]. Intravenous immunoglobulins were administered in only one case in combination with steroids, with a favorable outcome [26]. Plasmapheresis was performed in one case with a favorable outcome, in a patient with hearing loss, who failed to show improvement after steroids [14]. In most cases, hearing improved gradually within days or weeks, whereas complete resolution of symptoms was reported in three cases [19,21].

## 5. Discussion

This paper presents a case of severe sensorineural hearing loss in the context of a relapse in a patient with AQP4-positive NMOSD for 25 years. The absence of waves I to V in ABRs from the right, as well as the absence of a lesion in the proximity to the cochlear nuclei on MRI in the present case, points to a lesion in the right peripheral auditory pathway (cochlea and/or auditory nerve), which is rare in NMOSD, an inflammatory astrocytopathy mainly affecting the CNS. Indeed, acute or subacute sensorineural hearing loss can be a symptom of multiple sclerosis [27]; however, this is a rather rare symptom in NMOSD, occurring in 1–3.3% of NMOSD patients [22,28]. Most cases exhibiting sudden hearing loss in the context of NMOSD relapse were AQ4-positive [14,15,16,17,18,20,22,26]. Although AQP4 titer is not predictive of relapses in NMOSD [29,30], one case reported increased AQP4 titer before each attack of hearing loss on each side [16]. Isolated hearing loss was only reported in two cases [19,20], whereas in the majority of cases it was accompanied by other symptoms such as tinnitus, ataxia, intractable nausea/vomiting, optic neuritis, and myelitis (in descending order) [14,15,16,17,22]. The severity of hearing loss ranged from mild to deafness [14,19], as in the present case. Hearing loss was more severe at higher frequencies [15,17,20,22]. Sudden hearing loss was the presenting symptom of NMOSD in many cases [14,15,18,26]. Bilateral affection of the auditory pathway, i.e., tinnitus and/or hearing loss at different degrees, occurred in many cases [14,15,19,22,31]. This pattern of (hearing) affection, starting on one side and subsequently affecting the other, can be, indeed, suggestive of autoimmune etiology [16].

Based on imaging or neurophysiological data (auditory brainstem responses, distortion product otoacoustic emissions, transient evoked otoacoustic emissions) from case studies so far [14,16,18,19], the auditory pathway impairment seems to be localized either centrally or peripherally. In the first case, the location of the lesion can affect either the cochlear nuclei [19] or higher levels in the brainstem, i.e., the area between the midbrain and pons [14]. The cochlear nuclei are located in the dorsal medulla, in an area adjacent to the fourth ventricle, which highly expresses aquaporin-4 and constitutes a target region in NMOSD [23]. In fact, the presence of AQP4 in the glia limitans of the ventral cochlear nucleus has been demonstrated through Western blotting and immunohistochemistry [32]. AQP4 antibodies from peripheral blood gain access to the CNS through a disturbed BBB, bind to AQP4 in astrocyte endfeet, leading to activation of complement and attraction of neutrophils, eosinophils, microglia, and T cells as well as IL-6 secretion by astrocytes, which further induces inflammation and BBB breakdown [33]. Hearing loss has been reported in other types of autoimmune encephalitis as well, such as in Kelch-like protein-11 [34,35,36], NMDA [37,38], LGI1 [39], GABA_A_ [40], and GFAP encephalitis [41]. In particular, hearing loss with tinnitus, accompanied by ataxia, is a characteristic feature in Kelch-like protein-11 encephalitis, which can occur in up to 45% of patients [34,42], and can even precede other neurological symptoms by months [42]. The mechanisms behind hearing loss in autoimmune encephalitis may include disturbance of the BBB [33,43], which allows the entrance of immune cells/autoantibodies against neuronal receptors or synaptic proteins in the CNS under the influence of inflammatory cytokines [43]. BBB disturbance occurs in many regions of the CNS, especially in the brainstem and temporal lobes, which are part of the auditory pathway, independent of the presence of lesions on MRI [36,44]. Another mechanism may be potassium or calcium dysregulation, especially in the cases of autoimmune encephalitis associated with NMDA, LGI1, Casp2, AMPAR, or DPPX antibodies [45,46,47].

**Figure 5:**
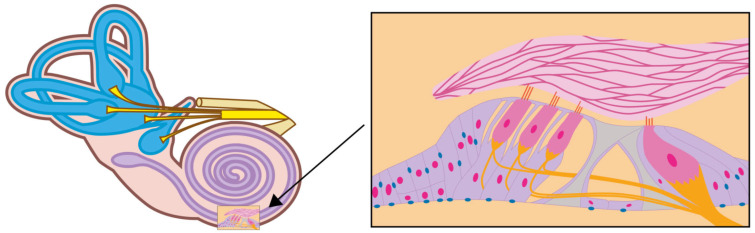
Schematic diagram depicting the localization of aquaporin-4 (blue dots) in the basolateral membrane of epithelial cells in the organ of Corti in the cochlea of the inner ear.

**Figure 6:**
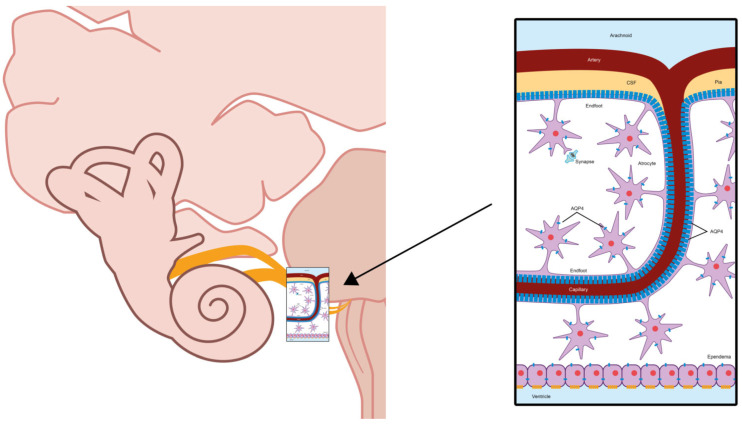
Schematic diagram depicting the localization of aquaporin-4 (blue cylinders) in the astrocytes, central to the central nervous system-peripheral nervous system transition of the cochlear and vestibular nerves.

In the case of affection of the peripheral auditory pathway, the impairment seems to be localized in the cochlear nerve itself, with enhancement of the nerve and adjacent meninges, as reported in one case [17] or in the cochlear cells of the inner ear, as reported in another case [16]. AQ4 is expressed in epithelial cells in the organ of Corti in the cochlea of the inner ear, as well as in the astrocytes central to the central nervous system (CNS)-peripheral nervous system transition of the cochlear and vestibular nerves, as previously shown in animal [48,49,50] and human studies [11] (Figure 5 and Figure 6). More precisely, AQ4 water channels are located at the basal or basolateral membrane of the supporting cells of the inner and outer sulcus, Hensen’s cells, and Claudius cells [11,51]. Notably, AQP4 is expressed in other sites outside the CNS as well, i.e., in the central components of spinal nerve roots and the cauda equina adjacent to the spinal cord [52,53,54].

Aquaporins are transmembrane channel proteins that exert a crucial role in regulating the volume and internal osmotic pressure of cells [55]. AQP4 plays a major role in fluid homeostasis and potassium recycling into the cochlea [50,56]. It maintains an osmotic gradient favoring potassium diffusion through the basolateral membrane of supporting cells of the organ of Corti [51]. This perilymphatic-endolymphatic potassium recycling contributes to the maintenance of the high potassium concentration in the endolymph that is critical to excitability and function of inner cells and necessary for transduction of sounds into electrical signals [57,58,59]. Therefore, sensorineural hearing loss may occur due to disruption of the ionic homeostasis of the cochlear potassium recycling network, resulting in hair cell dysfunction and cochlear cell toxicity [60]. The way AQP4-IgG antibodies reach the inner ear remains to be elucidated; however, it could be hypothesized that they pass from the plasma to the inner ear through the blood-perilymph barrier, in the same way they pass the BBB [56]. Therefore, a possible explanation for the early manifestation of NMOSD as hearing loss could be a break in immune tolerance to AQP4 located outside the CNS, such as in the inner ear, retrocochlear locations, or marginal sites between the peripheral nervous system and CNS, at the early disease stages [21]. Inflammation of the CNS with BBB leakage can follow; especially infratentorial structures, rich in AQ4, can be early affected [21]. The hearing impairment in NMOSD might also be associated with inflammation of the inner ear; i.e., AQP4 antibodies likely cause a form of “labyrinthitis” [48]. Another hypothesis is that of a “focal astrocytopathy”, as the supportive cells of the inner ear may carry on functions similar to those of astrocytes in the CNS [48].

Animal studies showed that knocking out/blocking AQP4 in the organ of Corti and cochlear nerve may inhibit its osmoregulatory function, resulting in hearing loss [61]. AQP4-deficient mice have hearing impairments of varying severity, up to the point of deafness, as revealed by the analysis of the auditory brainstem threshold [49,50]. On the other hand, mice deficient in AQP1, AQP3, and AQP5 have no hearing impairment [49]. AQP4 knockout mice showed impaired hearing but normal neural conduction time, suggesting cochlear dysfunction as the primary cause of hearing loss [50]. The hearing impairment in AQP4-deficient mice can be attributed to an AQP4-dependent functional disturbance of the supporting cells of the cochlea expressing AQP4 water channels in their basal or basolateral membranes [56]. The type of hearing loss in these mice is sensory, not neural, and is therefore cochlear and not retrocochlear [50].

ABRs were performed in many cases, aiding the diagnosis of a central disorder, even in the absence of characteristic brainstem lesions on MRI [14,18,19]. In the present case, the absence of waves I to V in ABRs from the right points to a lesion in the right peripheral auditory pathway (cochlea and/or auditory nerve). Literature data are quite controversial regarding the contribution of auditory evoked potentials in the diagnosis of NMOSD [62]; however, a recent study demonstrated abnormal ABRs in 33% of NMOSD patients, with increased I–III interpeak latency being the most prevalent finding [63]. Abnormal ABRs in the absence of lesions on brain imaging could be due to lesions not being visible on MRI or reduced brainstem volume, i.e., brainstem atrophy, which is common in NMOSD patients [64].

Hearing loss was sensorineural in the majority of reviewed cases (60%). On the other hand, conductive hearing loss has only been reported in one case of seropositive NMOSD [15]. Conductive hearing loss has been reported in other autoimmune diseases such as lupus erythematosus or various vasculitides [65]. Indeed, autoimmune disorders can induce conductive hearing loss as a result of various mechanisms such as effusions of the middle ear, inflammation of the mucosa of the Eustachian tube, or involvement of the ossicular chain [65].

The patients with NMOSD exhibiting hearing loss were treated in most cases with high-dose steroids intravenously or orally, usually followed by oral tapering up to two months. Plasmapheresis [14] or intravenous immunoglobulins [26] were reported in one case each, in combination with steroids, with a positive outcome. A recent meta-analysis on the efficacy of plasmapheresis in acute attacks of NMOSD demonstrated that it is an effective therapeutic method during acute attacks, whether used as rescue therapy when intravenous corticosteroids are insufficient, or as a first-line treatment [66]. Earlier initiation of plasmapheresis is usually associated with greater benefit [66]. In the vast majority of cases, treatment resulted in gradual, significant improvement or resolution of hearing symptoms within days or weeks. In our case, there was a partial recovery of sensorineural hearing loss within three months. Delayed recognition and treatment may have a negative impact on hearing outcome, as reported previously [16].

## 6. Conclusions

Autoimmune sensorineural hearing loss accounts for less than 1% of hearing loss causes, with both humoral and cell-mediated mechanisms involved in the impairment [67]. Clinical features in immune-mediated sensorineural hearing loss consist of unexplained, unilateral or often bilateral hearing loss, rapid progression over days or weeks, steroid-responsiveness, and coexistence with systemic immune disorders [12]. Relapses of NMOSD with symptoms such as tinnitus or mild hearing loss may be overlooked, resulting in patient morbidity. It is also possible that auditory (peripheral) damage is more common than currently thought in NMOSD. Emphasis should be given to additional signs such as ocular movements, i.e., an abnormal Head-impulse-test (HIT) when the immune attack involves the vestibular nerve or the direct pathway of the vestibular ocular reflex [68]. The above also stress the need for objective examination of auditory symptoms with verified methods such as audiometry, caloric test with electro-/video-nystagmographic recordings, vestibular evoked myogenic potentials (when available), and ABRs [12]. Timely recognition of NMOSD relapse and treatment is crucial to prevent permanent loss of function of sensory organs such as vision and hearing.

This paper describes a rare case of peripheral auditory pathway affection in NMOSD, which is an inflammatory astrocytopathy mainly affecting the CNS. This paper expands our knowledge of overlapping central and peripheral nervous system inflammatory syndromes [69]. AQP4 antibodies can cause damage to peripheral organs beyond the CNS, such as vestibulocochlear nerves, muscle, gastrointestinal tract, kidney, blood system, lung, and placenta [55,61,70]. For this reason, the term “autoimmune aquaporin-4 channelopathy” has been proposed to incorporate various central and peripheral disorders associated with AQP4 antibodies [55]. The degree of injury of the peripheral organs seems to be less severe than the CNS, which may be related to the different distributions of AQP4 isoforms and the different number of complement regulatory factors expressed in various organs [48]. The above, along with the growing knowledge of AQP4 immunopathogenic mechanisms, further expands the disease spectrum of autoimmune aquaporin-4 channelopathy. Future studies can assess if aquaporins can constitute therapeutic targets for the treatment of inner ear diseases.

## Figures and Tables

**Table 1:** Clinical information of the presented case.

**Demographics**	58-Year-Old Female
**Presenting symptoms**	vertigo, tinnitus, loss of hearing in the right ear, gait instability
**Medical history**	aquaporin-4-positive NMOSD, hypertension, hyperlipidemia
**Neurological examination**	severe truncal ataxia, severe hearing loss in the right ear absent pupillary light reflex on the left and mild left spastic hemiparesis with hemihypesthesia (residual)
**Investigations**	Brain MRI: multiple non-enhancing periventricular and subcortical lesions, lesions in the pons Pure-tone audiogram: hearing threshold at 90–100 dBnHL at all frequencies, compatible with deafness on the right ABRs: on the right no waves I-V could be recorded, normal on the left
**Treatment**	1 g methylprednisolone/day for 5 days -> no improvement of hearing 3 intratympanic steroid injections (dexamethasone 4 mg each) -> no improvement in hearing plasmapheresis q.o.d. for 14 days -> slight improvement of hearing
**Outcome/Follow-up**	3-month follow-up: moderate hearing levels (45–55 dBnHL) at low frequencies to severe hearing levels (75–95 dBnHL) at high frequencies on the right ear

Abbreviations: NMOSD: neuromyelitis optica spectrum disorder, MRI: Magnetic resonance imaging, ABRs: Auditory brainstem responses, q.o.d.: every other day.

**Table 2:** Previous cases of patients with hearing loss due to NMOSD.

Authors, Year	Age	Gender	Symptoms	Type of Hearing Loss	MRI Lesions	Audiogram	Neurophysiology	AQ4 Antibodies	Treatment	Outcome
**Onda et al., 2021** [14]	49	female	Diplopia, bilateral ptosis, downgaze palsy, truncal ataxia, bilateral tinnitus, deafness	Sensorineural	midbrain, pons, dorsal medulla, around the fourth ventricle	n/a	ABR potentials: no wave V on left stimulation, and extended wave III-V latency on right stimulation	positive	Four courses of MP 1 g/d for 3 days each, followed by 20 mg oral prednisolone and PLEX	hearing loss improved gradually after PLEX
**Tugizova et al., 2020** [15] Case 1	54	female	Intractable vomiting, hiccups, vertigo, bilateral hearing loss, tinnitus	Sensorineural	cervico-medullary junction	mild to moderate sensorineural hearing loss at 3000–8000 Hz in the right ear, at 6000–8000 Hz in left ear	n/a	positive	MP iv., followed by a 6-week prednisone tapering	Full recovery of hearing
Case 2	26	female	Left-sided hearing loss, tinnitus, recurrent left optic neuritis	Conductive	White matter lesions	mild conductive hearing loss at low frequencies or bony hyperacusis on the left side	n/a	positive	High-dose steroids, rituximab	Hearing loss and tinnitus persisted
**Shaw et al. 2021** [16]	54	female	Right-sided tinnitus, bilateral hearing loss	Sensorineural	n/a	moderate, low, and mid-frequency right-sided hearing loss, mild low-frequency left-sided hearing loss	Left-sided vestibular hypofunction Transient evoked otoacoustic emissions absent on the right ABR, cervical vestibular-evoked myogenic potentials: normal	positive	5 days of oral MP 500 mg, followed by a 2-month tapering	Left-sided hearing loss normalized, right-sided unchanged
**Bonnan et al. 2017** [17]	53	female	Right-sided hearing loss, tinnitus, vertigo	Sensorineural	Enhancement and enlargement of the eighth cranial nerve and adjacent meninges	Right-sided hearing loss of 30 db in the higher frequencies	n/a	positive	High-dose steroids, followed by six infusions of mitoxantrone over one year	1-year follow-up: almost normal auditory function, MRI normalized
**Takanashi et al. 2014** [18]	40	female	Diplopia, hemianopsia, numbness in hands and feet, dysuria, right-sided hearing loss	Sensorineural	optic chiasm, optic tract, hypothalamus, cerebral fornix, cervical cord	Minimal difference in pure tone threshold on the right	ABR severely attenuated in the right ear Distortion product otoacoustic emission normal	positive	Two courses of MP pulse therapy iv. (1 g/d for 3 days each), followed by oral tapering for 28 days	Improvement of hearing loss
**Gratton et al. 2014** [19]	54	female	Bilateral hearing loss progressing to deafness over 2 days	Sensorineural	near the cochlear nuclei	n/a	ABR and otoacoustic emission testing consistent with central lesions bilaterally	n/a	MP intravenous	Resolution of symptoms
**Jarius et al. 2013** [20]	51	male	Acute left-sided hearing loss	n/a	No new lesions	n/a	n/a	yes	Prednisolone 100 mg/d, followed by oral tapering to 10 mg/d	Improvement within 10 days, 1-month follow-up: normal hearing
**Kremer et al. 2013** [21] 3 patients with hearing loss out of 258 NMOSD patients	44.2 mean age	n/a	Hearing loss	n/a	n/a, no correlation with MRI	n/a	n/a	One positive, two negative	n/a	Two recovered completely, one with persistent sequelae
**Cai et al. 2024** [26]	34	female	Vomiting, dysphagia, diplopia, hearing loss, left-sided facial palsy, breathing difficulties, hoarseness of voice	Sensorineural	medulla oblongata, left middle cerebellar peduncle	n/a	n/a	positive	MP 1 g/d iv. and immunoglobulins 0.4 mg/kg for five days, followed by oral tapering	3-month follow-up: significant improvement
**Kwon et al. 2024** [22] Case 1	49	female	Dizziness, bilateral hearing loss	Sensorineural	pons, around fourth ventricle, left lateral medulla	bilateral sensorineural hearing loss	n/a	positive	Oral prednisolone	No recovery of hearing
Case 2	70	female	Vertigo, ear fullness, tinnitus, right-sided hearing loss, left-beating nystagmus	Mixed-type	Corpus callosum lesion	Mixed-type hearing loss	n/a	positive	High-dose MP iv.	No improvement of hearing (rest of symptoms improved)
Case 3	34	female	Bilateral hearing loss at age 26	Sensorineural	No new lesions compared to the previous	bilateral sensorineural hearing loss at high frequency	n/a	positive	10 mg oral prednisolone q.o.d.	No improvement of hearing

Abbreviations: MRI: magnetic resonance imaging, AQ4: aquaporin-4, ABR: Auditory brainstem response, MP: methylprednisolone, iv.: intravenously, PLEX: plasma exchange/plasmapheresis, n/a: not reported.

## Data Availability

The data that support the findings of this study are available from the corresponding author upon reasonable request.

## Supplementary Materials

The following supporting information can be downloaded at: https://www.mdpi.com/article/10.3390/jcm15020422/s1. Supplementary Figure S1: Auditory evoked potentials (a) from the left ear and (b) from the right ear. Supplementary Figure S2. Pure-tone audiogram after completion of plasmapheresis showing severe sensorineural hearing loss at all frequencies, except for 8 kHz, where the hearing threshold was at 105 dBnHL. PRISMA checklist: PRISMA 2020 for Abstracts Checklist, Ref. [71] is listed in the supplementary.

## References

[1] Jarius S., Aktas O., Ayzenberg I., Bellmann-Strobl J., Berthele A., Giglhuber K., Häußler V., Havla J., Hellwig K., Hümmert M.W. (2023). Update on the diagnosis and treatment of neuromyelits optica spectrum disorders (NMOSD)—Revised recommendations of the Neuromyelitis Optica Study Group (NEMOS). Part I: Diagnosis and differential diagnosis. J. Neurol..

[2] Mangiatordi G.F., Alberga D., Trisciuzzi D., Lattanzi G., Nicolotti O. (2016). Human Aquaporin-4 and Molecular Modeling: Historical Perspective and View to the Future. Int. J. Mol. Sci..

[3] Carnero Contentti E., Correale J. (2021). Neuromyelitis optica spectrum disorders: From pathophysiology to therapeutic strategies. J. Neuroinflamm..

[4] Jarius S., Paul F., Franciotta D., Waters P., Zipp F., Hohlfeld R., Vincent A., Wildemann B. (2008). Mechanisms of disease: Aquaporin-4 antibodies in neuromyelitis optica. Nat. Clin. Pr. Pract. Neurol..

[5] Ala M., Mohammad Jafari R., Hajiabbasi A., Dehpour A.R. (2021). Aquaporins and diseases pathogenesis: From trivial to undeniable involvements, a disease-based point of view. J. Cell Physiol..

[6] Nagelhus E.A., Ottersen O.P. (2013). Physiological roles of aquaporin-4 in brain. Physiol. Rev..

[7] Ikeshima-Kataoka H. (2016). Neuroimmunological Implications of AQP4 in Astrocytes. Int. J. Mol. Sci..

[8] Ximenes-da-Silva A., Capra D., Sanz C.K., Mendes C.B., de Mattos Coelho Aguiar J., Moura-Neto V., DosSantos M.F. (2022). The role of aquaporins in hearing function and dysfunction. Eur. J. Cell Biol..

[9] Dong S.H., Kim S.S., Kim S.H., Yeo S.G. (2020). Expression of aquaporins in inner ear disease. Laryngoscope.

[10] Huang D., Chen P., Chen S., Nagura M., Lim D.J., Lin X. (2002). Expression patterns of aquaporins in the inner ear: Evidence for concerted actions of multiple types of aquaporins to facilitate water transport in the cochlea. Hear. Res..

[11] Lopez I.A., Ishiyama G., Lee M., Baloh R.W., Ishiyama A. (2007). Immunohistochemical localization of aquaporins in the human inner ear. Cell Tissue Res..

[12] Young Y.H. (2020). Contemporary review of the causes and differential diagnosis of sudden sensorineural hearing loss. Int. J. Audiol..

[13] Prince A.D.P., Stucken E.Z. (2021). Sudden Sensorineural Hearing Loss: A Diagnostic and Therapeutic Emergency. J. Am. Board. Fam. Med..

[14] Onda A., Yamazaki M., Shimoyama T., Yaguchi H. (2021). Neuromyelitis optica spectrum disorder with deafness and an extensive brainstem lesion. Heliyon.

[15] Tugizova M., Feng H., Tomczak A., Steenerson K., Han M. (2020). Case series: Hearing loss in neuromyelitis optica spectrum disorders. Mult. Scler. Relat. Disord..

[16] Shaw B., Raghavan R.S., Warner G., Palace J. (2021). ‘Cochlear-type’ hearing loss as part of aquaporin-4 neuromyelitis optica spectrum disorder. BMJ Case Rep..

[17] Bonnan M., Cabre P. (2017). Meningeal and vestibulocochlear nerve enhancement in neuromyelitis optica. Eur. J. Neurol..

[18] Takanashi Y., Misu T., Oda K., Miyazaki H., Yahata I., Hidaka H., Fujihara K., Kawase T., Kobayashi T., Katori Y. (2014). Audiological evidence of therapeutic effect of steroid treatment in neuromyelitis optica with hearing loss. J. Clin. Neurosci. Off. J. Neurosurg. Soc. Australas..

[19] Gratton S., Amjad F., Ghavami F., Osborne B., Tornatore C., Mora C. (2014). Bilateral hearing loss as a manifestation of neuromyelitis optica. Neurology.

[20] Jarius S., Lauda F., Wildemann B., Tumani H. (2013). Steroid-responsive hearing impairment in NMO-IgG/aquaporin-4-antibody-positive neuromyelitis optica. J. Neurol..

[21] Kremer L., Mealy M., Jacob A., Nakashima I., Cabre P., Bigi S., Paul F., Jarius S., Aktas O., Elsone L. (2014). Brainstem manifestations in neuromyelitis optica: A multicenter study of 258 patients. Mult. Scler..

[22] Kwon S.C.S., Chung Y.H., Min J.-H. (2024). Sensorineural Hearing Loss in Seropositive Neuromyelitis Optica Spectrum Disorder and Myelin Oligodendrocyte Glycoprotein Antibody-Associated Disorder. Acta Neurol. Scand..

[23] Pittock S.J., Weinshenker B.G., Lucchinetti C.F., Wingerchuk D.M., Corboy J.R., Lennon V.A. (2006). Neuromyelitis optica brain lesions localized at sites of high aquaporin 4 expression. Arch. Neurol..

[24] Tricco A.C., Lillie E., Zarin W., O’Brien K.K., Colquhoun H., Levac D., Moher D., Peters M.D.J., Horsley T., Weeks L. (2018). PRISMA Extension for Scoping Reviews (PRISMA-ScR): Checklist and Explanation. Ann. Intern. Med..

[25] Ma L.L., Wang Y.Y., Yang Z.H., Huang D., Weng H., Zeng X.T. (2020). Methodological quality (risk of bias) assessment tools for primary and secondary medical studies: What are they and which is better?. Mil Med. Res..

[26] Cai L., Liu X., Zhou H., Li J., Zhou D., Hong Z. (2024). Case report: Identification of Hepatitis B Virus in the cerebrospinal fluid of neuromyelitis optica spectrum disorders and successful treatment with ofatumumab and inebilizumab. Front. Immunol..

[27] Mirmosayyeb O., Naderi M., Raeisi S., Ebrahimi N., Ghaffary E.M., Afshari-Safavi A., Barzegar M., Shaygannejad V. (2022). Hearing loss among patients with multiple sclerosis (PwMS): A systematic review and meta-analysis. Mult. Scler. Relat. Disord..

[28] Tanaka M., Tanaka K. (2016). Sudden hearing loss as the initial symptom in Japanese patients with multiple sclerosis and seropositive neuromyelitis optica spectrum disorders. J. Neuroimmunol..

[29] Jitprapaikulsan J., Fryer J.P., Majed M., Smith C.Y., Jenkins S.M., Cabre P., Hinson S.R., Weinshenker B.G., Mandrekar J., Chen J.J. (2020). Clinical utility of AQP4-IgG titers and measures of complement-mediated cell killing in NMOSD. Neurol. Neuroimmunol. Neuroinflamm..

[30] Kessler R.A., Mealy M.A., Jimenez-Arango J.A., Quan C., Paul F., López R., Hopkins S., Levy M. (2017). Anti-aquaporin-4 titer is not predictive of disease course in neuromyelitis optica spectrum disorder: A multicenter cohort study. Mult. Scler. Relat. Disord..

[31] Castillo-Torres S.A., Soto-Rincón C.A., Villarreal-Montemayor H.J., Chávez-Luévanos B. (2020). Case of neuromyelitis optica: Bilateral sensorineural hearing loss and transverse myelopathy following intrathecal chemotherapy. BMJ Case Rep..

[32] Hubbard J.A., Hsu M.S., Seldin M.M., Binder D.K. (2015). Expression of the Astrocyte Water Channel Aquaporin-4 in the Mouse Brain. ASN Neuro.

[33] Shimizu F., Nakamori M. (2024). Blood-Brain Barrier Disruption in Neuroimmunological Disease. Int. J. Mol. Sci..

[34] Vogrig A., Péricart S., Pinto A.L., Rogemond V., Muñiz-Castrillo S., Picard G., Selton M., Mittelbronn M., Lanoiselée H.M., Michenet P. (2021). Immunopathogenesis and proposed clinical score for identifying Kelch-like protein-11 encephalitis. Brain Commun..

[35] Mandel-Brehm C., Dubey D., Kryzer T.J., O’Donovan B.D., Tran B., Vazquez S.E., Sample H.A., Zorn K.C., Khan L.M., Bledsoe I.O. (2019). Kelch-like Protein 11 Antibodies in Seminoma-Associated Paraneoplastic Encephalitis. N. Engl. J. Med..

[36] Gilligan M., Thakolwiboon S., Orozco E., Banks S., Flanagan E.P., Lopez-Chiriboga S., Tillema J.M., Mills J.R., Pittock S.J., Valencia Sanchez C. (2025). Autoimmune brainstem encephalitis: Clinical associations, outcomes, and proposed diagnostic criteria. Ann. Clin. Transl. Neurol..

[37] Cheng H., Yang F., Zhang J., Xu L., Jia L., Zhao D., Liu W., Li H. (2021). Case Report: Anti-NMDA Receptor Encephalitis With Bilateral Hearing Loss as the Initial Symptom. Front. Neurol..

[38] Zhang G.F., Liang T., Lv Y.K., Luo Z., Zhang J. (2024). Bilateral hearing loss caused by anti-NMDA receptor encephalitis with teratoma: A case report. Ibrain.

[39] Saleem N., Robbins M., Foster J., Kelley B. (2025). Hearing Loss in Leucine-Rich Glioma-Inactivated 1 Encephalitis: Cochlear Implantation Considerations. Cureus.

[40] Boisclair M., Robitaille C., Budhram A., Kunchok A., Chapdelaine H., Létourneau-Guillon L., Macaron G., Larochelle C. (2024). Severe Relapsing Autoimmune Encephalitis with GABA(A) Receptor, Titin, and AchR Antibodies in a Patient with Thymoma: A Case Report. Case Rep. Neurol..

[41] Ip B., Lam C., Ip V., Chan A., Mok V., Au E., Chan E., Lau A. (2020). Autoimmune glial fibillary acidic protein astrocytopathy associated meningoencephalomyelitis and bilateral sensorineuronal deafness. Mult. Scler. Relat. Disord..

[42] Dubey D., Wilson M.R., Clarkson B., Giannini C., Gandhi M., Cheville J., Lennon V.A., Eggers S., Devine M.F., Mandel-Brehm C. (2020). Expanded Clinical Phenotype, Oncological Associations, and Immunopathologic Insights of Paraneoplastic Kelch-like Protein-11 Encephalitis. JAMA Neurol..

[43] Platt M.P., Agalliu D., Cutforth T. (2017). Hello from the Other Side: How Autoantibodies Circumvent the Blood-Brain Barrier in Autoimmune Encephalitis. Front. Immunol..

[44] Filippi M., Rocca M.A. (2024). Autoimmune Encephalitis and Blood-Brain Barrier Permeability at Dynamic Contrast-enhanced MRI. Radiology.

[45] Schmaul S., Hanuscheck N., Bittner S. (2021). Astrocytic potassium and calcium channels as integrators of the inflammatory and ischemic CNS microenvironment. Biol. Chem..

[46] Baraibar A.M., Colomer T., Moreno-García A., Bernal-Chico A., Sánchez-Martín E., Utrilla C., Serrat R., Soria-Gómez E., Rodríguez-Antigüedad A., Araque A. (2024). Autoimmune inflammation triggers aberrant astrocytic calcium signaling to impair synaptic plasticity. Brain Behav. Immun..

[47] Ryding M., Mikkelsen A.W., Nissen M.S., Nilsson A.C., Blaabjerg M. (2023). Pathophysiological Effects of Autoantibodies in Autoimmune Encephalitides. Cells.

[48] Takumi Y., Nagelhus E.A., Eidet J., Matsubara A., Usami S., Shinkawa H., Nielsen S., Ottersen O.P. (1998). Select types of supporting cell in the inner ear express aquaporin-4 water channel protein. Eur. J. Neurosci..

[49] Li J., Verkman A.S. (2001). Impaired hearing in mice lacking aquaporin-4 water channels. J. Biol. Chem..

[50] Mhatre A.N., Stern R.E., Li J., Lalwani A.K. (2002). Aquaporin 4 expression in the mammalian inner ear and its role in hearing. Biochem. Biophys. Res. Commun..

[51] Mack A.F., Wolburg H. (2013). A novel look at astrocytes: Aquaporins, ionic homeostasis, and the role of the microenvironment for regeneration in the CNS. Neuroscientist.

[52] Grüter T., Ayzenberg I., Gahlen A., Kneiphof J., Gold R., Kleiter I. (2018). Flaccid paralysis in neuromyelitis optica: An atypical presentation with possible involvement of the peripheral nervous system. Mult. Scler. Relat. Disord..

[53] Takai Y., Misu T., Nakashima I., Takahashi T., Itoyama Y., Fujihara K., Aoki M. (2012). Two cases of lumbosacral myeloradiculitis with anti-aquaporin-4 antibody. Neurology.

[54] Kim S., Park J., Kwon B.S., Park J.W., Lee H.J., Choi J.H., Nam K. (2017). Radiculopathy in neuromyelitis optica. How does anti-AQP4 Ab involve PNS?. Mult. Scler. Relat. Disord..

[55] He D., Zhang A., Li Y., Cai G., Li Y., Guo S. (2017). Autoimmune aquaporin-4 induced damage beyond the central nervous system. Mult. Scler. Relat. Disord..

[56] Eckhard A., Gleiser C., Arnold H., Rask-Andersen H., Kumagami H., Müller M., Hirt B., Löwenheim H. (2012). Water channel proteins in the inner ear and their link to hearing impairment and deafness. Mol. Asp. Med..

[57] Wangemann P. (2006). Supporting sensory transduction: Cochlear fluid homeostasis and the endocochlear potential. J. Physiol..

[58] Hibino H., Nin F., Tsuzuki C., Kurachi Y. (2010). How is the highly positive endocochlear potential formed? The specific architecture of the stria vascularis and the roles of the ion-transport apparatus. Pflug. Arch..

[59] Nin F., Hibino H., Doi K., Suzuki T., Hisa Y., Kurachi Y. (2008). The endocochlear potential depends on two K^+^ diffusion potentials and an electrical barrier in the stria vascularis of the inner ear. Proc. Natl. Acad. Sci. USA.

[60] Christensen N., D’ouza M., Zhu X., Frisina R.D. (2009). Age-related hearing loss: Aquaporin 4 gene expression changes in the mouse cochlea and auditory midbrain. Brain Res..

[61] Noori H., Marsool M.D.M., Gohil K.M., Idrees M., Subash T., Alazzeh Z., Prajjwal P., Jain H., Amir O. (2024). Neuromyelitis optica spectrum disorder: Exploring the diverse clinical manifestations and the need for further exploration. Brain Behav..

[62] Ohnari K., Okada K., Takahashi T., Mafune K., Adachi H. (2016). Evoked potentials are useful for diagnosis of neuromyelitis optica spectrum disorder. J. Neurol. Sci..

[63] Mielle L.P., Silva L.A.F., Barbosa D.A.N., Martins G.A., Samelli A.G., Matas C.G. (2025). Evaluation of Auditory Evoked Potentials in Neuromyelitis Optica Spectrum Disorder. J. Integr. Neurosci..

[64] Lee C.Y., Mak H.K., Chiu P.W., Chang H.C., Barkhof F., Chan K.H. (2018). Differential brainstem atrophy patterns in multiple sclerosis and neuromyelitis optica spectrum disorders. J. Magn. Reson. Imaging.

[65] Rahne T., Plontke S., Keyßer G. (2020). Vasculitis and the ear: A literature review. Curr. Opin. Rheumatol..

[66] Yu H.H., Qin C., Zhang S.Q., Chen B., Ma X., Tao R., Chen M., Chu Y.H., Bu B.T., Tian D.S. (2021). Efficacy of plasma exchange in acute attacks of neuromyelitis optica spectrum disorders: A systematic review and meta-analysis. J. Neuroimmunol..

[67] Mijovic T., Zeitouni A., Colmegna I. (2013). Autoimmune sensorineural hearing loss: The otology-rheumatology interface. Rheumatology.

[68] Shaikh A.G., Manto M. (2019). Distillation of Posterior Fossa Demyelination in Acute Vestibular Syndrome: The Eyes Have It. Cerebellum.

[69] Rinaldi S., Davies A., Fehmi J., Beadnall H.N., Wang J., Hardy T.A., Barnett M.H., Broadley S.A., Waters P., Reddel S.W. (2021). Overlapping central and peripheral nervous system syndromes in MOG antibody-associated disorders. Neurol. Neuroimmunol. Neuroinflamm..

[70] Rosales D., Kister I. (2016). Common and Rare Manifestations of Neuromyelitis Optica Spectrum Disorder. Curr. Allergy Asthma Rep..

[71] Page M.J., McKenzie J.E., Bossuyt P.M., Boutron I., Hoffmann T.C., Mulrow C.D., Shamseer L., Tetzlaff J.M., Akl E.A., Brennan S.E. (2021). The PRISMA 2020 statement: An updated guideline for reporting systematic reviews. BMJ.

