# Supplementary figures and images for: Hearing Loss in Neuromyelitis Optica Spectrum Disorder: Case Report and Systematic Review
Source: J Clin Med. 2026 Jan 6;15(2):422. doi: 10.3390/jcm15020422 (PMC12841620; doi:10.3390/jcm15020422)

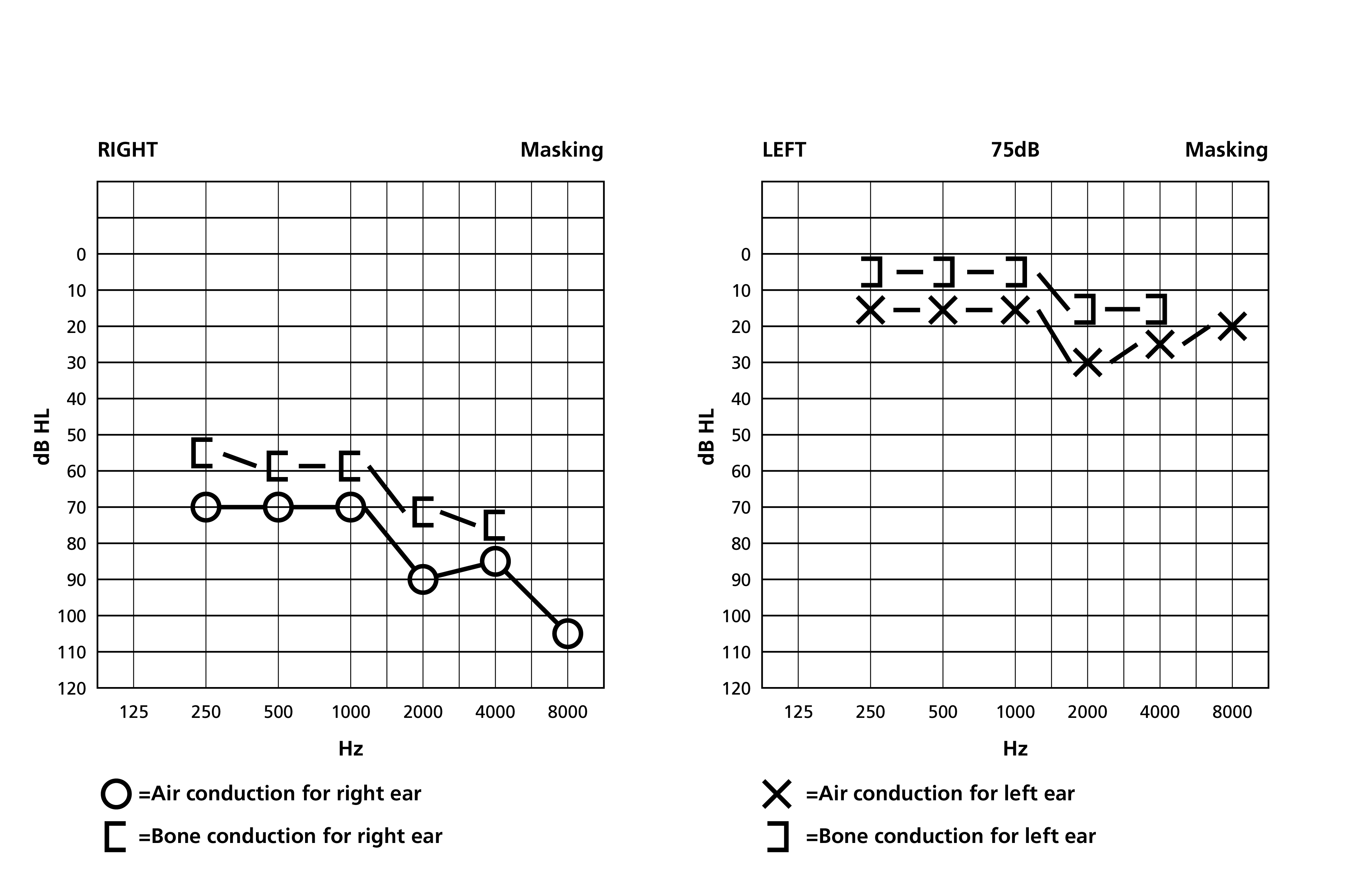

Supplement: Supplementary file 1 [file jcm-15-00422-s001.zip › After plasmapheresis figure suppl2.jpg]

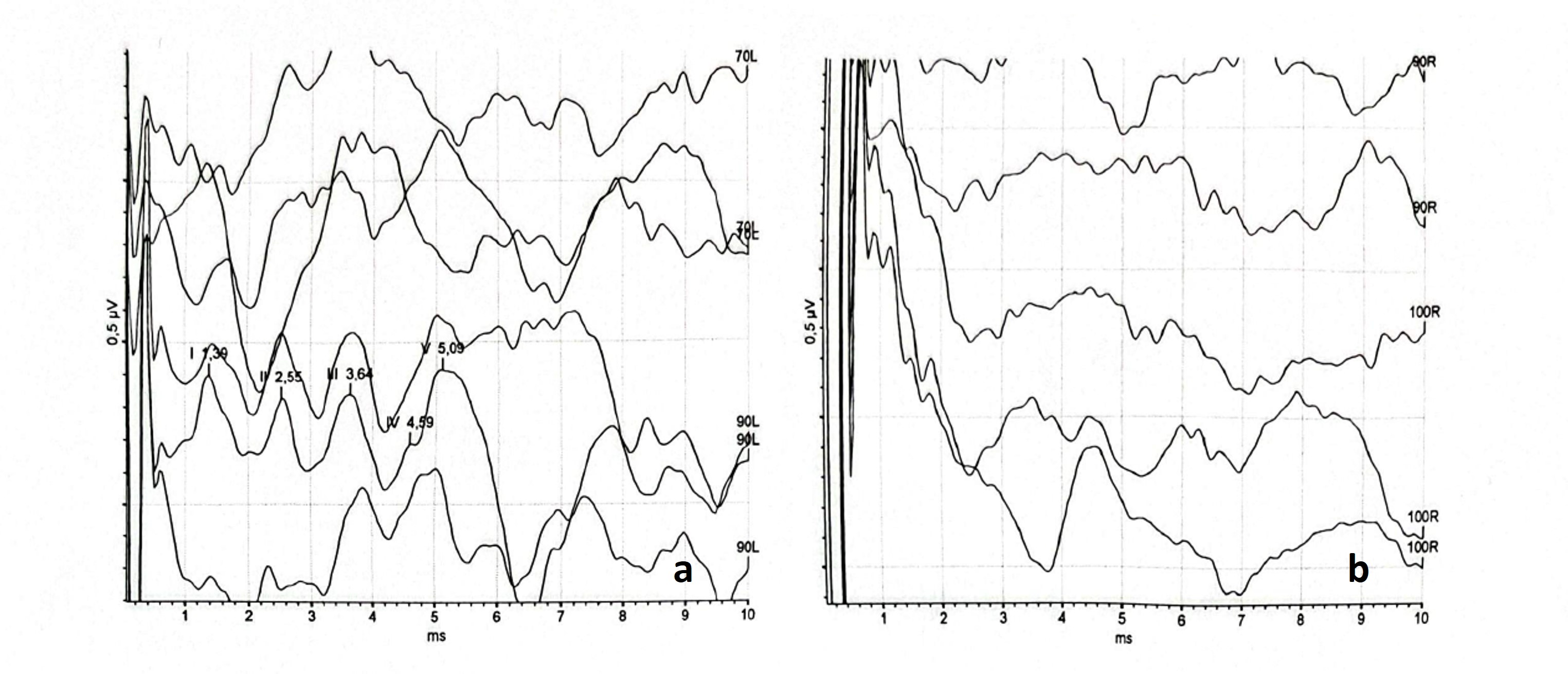

Supplement: Supplementary file 1 [file jcm-15-00422-s001.zip › figure evoked potentials suppl1.jpg]
